# Background climate modulates the impact of land cover on urban surface temperature

**DOI:** 10.1038/s41598-022-19431-x

**Published:** 2022-09-14

**Authors:** Marzie Naserikia, Melissa A. Hart, Negin Nazarian, Benjamin Bechtel

**Affiliations:** 1 Australian Research Council Centre of Excellence for Climate Extremes, University of New South Wales, Sydney, Australia; 2 School of Built Environment, University of New South Wales, Sydney, Australia; 3 City Futures Research Centre, University of New South Wales, Sydney, Australia; 4 Department of Geography, Ruhr-University Bochum, Bochum, Germany

**Keywords:** Climate sciences, Environmental sciences

## Abstract

Cities with different background climates experience different thermal environments. Many studies have investigated land cover effects on surface urban heat in individual cities. However, a quantitative understanding of how background climates modify the thermal impact of urban land covers remains elusive. Here, we characterise land cover and their impacts on land surface temperature (LST) for 54 highly populated cities using Landsat-8 imagery. Results show that urban surface characteristics and their thermal response are distinctly different across various climate regimes, with the largest difference for cities in arid climates. Cold cities show the largest seasonal variability, with the least seasonality in tropical and arid cities. In tropical, temperate, and cold climates, normalised difference built-up index (NDBI) is the strongest contributor to LST variability during warm months followed by normalised difference vegetation index (NDVI), while normalised difference bareness index (NDBaI) is the most important factor in arid climates. These findings provide a climate-sensitive basis for future land cover planning oriented at mitigating local surface warming.

## Introduction

With a rapidly urbanised world, adapting urban areas to local and global climate change is one of the most important global challenges. At the local scale, elevated urban temperature is the most well-known impact of urbanisation^1^, which can significantly influence citizens' health and well-being^2–4^ and increase energy consumption, greenhouse gas emissions, and anthropogenic heat in cities^5,6^. The urban heat island is often used to characterise this phenomenon, defined as the temperature difference between urban and surrounding rural areas and considered the additional heat released to the environment by cities^7,8^. However, recently, the accuracy of this representation has been disputed^9^. Urban areas unavoidably affect nearby rural areas; hence, it is difficult to determine an appropriate reference for analysing temperature deviations as the temperature observed in these areas differs from the temperature that would be experienced in the absence of cities. Therefore, instead of focusing on assessing the temperature difference between rural and urban areas, it is essential to understand the intra-urban temperature variability within the city to minimise the negative effects of urban overheating.

The conversion of natural land to built-up surfaces with distinct modification to urban form and fabric has been widely documented as the main determinant of warming across urban areas^10^. Impermeable and dark materials used in urban areas result in an absence of moisture to dissipate the heat from the sun and trapping more of the sun’s energy^11,12^. Thus, impervious surfaces favour sensible heat over latent heat in comparison to the previously vegetated areas, increasing temperature in cities. This land conversion process significantly influences both canopy and surface urban heat represented by air and land surface temperature (LST), respectively^13^. LST is a key factor determining the interaction of atmosphere with Earth’s surface^14^. Although there are many studies analysing the intensity and the spatial distribution of land cover properties and their impact on LST in various cities, uncertainties remain regarding how the surface characteristics of these land covers and their thermal effects vary across diverse climate regimes.

Background climate has a significant impact on surface urban heat^15–17^ as it can influence surface radiation and the energy flux between urban and non-urban areas. Two cities with very similar structure, morphology, land cover, materials, population, and anthropogenic activities may experience very different urban heat solely based on the characteristics of their background climates^9^. Several studies have found that the main cause of the temporal and spatial variability of surface urban heat is climate-vegetation background^18–20^. Vegetation with its evaporative cooling effect has been widely documented as one of the key factors in regulating urban surface temperature^19,21,22^. A global study of more than 400 cities, which investigated average annual, seasonal, and diurnal variations of Surface Urban Heat Island (SUHI) intensity (derived from MODIS), found no correlation between city area size or population density and SUHI intensity, but strong correlations with urban vegetation cover. It is also reported that vegetation evaporative cooling can significantly control the SUHI during the day^22^. However, vegetation cooling is affected by background climate as it changes the longwave radiation and the sensible heat flux and, therefore, can control the direction of heat transfer^23–25^.

The contribution of vegetation cover in the thermal performance of urban areas has been often investigated. However, studies that evaluate the role of other land cover types in cooling and heating cities with different climates are still scarce. Using the MODIS dataset, a recent study assessed the influence of the spatial configuration of different land covers on LST in seven large cities having different Köppen Geiger climate classes in the USA. They reported that the relationship between LST and spatial clustering of land use/land covers is significantly affected by the regional climate background of a city^26^. Although there are a few studies showing the importance of background climate on urban heating and cooling, they lack details regarding how the climate of a city can impact urban heat and how different land cover types contribute to intra-urban and inter-seasonal temperature variability in diverse climatic zones.

Land cover indices, such as NDBI, NDVI, NDBaI, and NDWI, aim to characterise the prevalence of built-up surfaces, vegetation cover, soil, and water bodies, respectively. The effect of land cover on surface urban heat has been often explored on a city-by-city basis by investigating the relationship between these land cover indices and LST. However, it is not possible to extrapolate their results beyond the city at hand, due to varying methodological approaches, different geographical locations and climatic conditions of the target cities. For example, the land cover indices significantly correlated to LST in some studies^27^ have been shown to have little correlation in others^28^. Therefore, some parameters may be identified as significant indicators of LST variability in some situations, while the same parameters may be less important in other conditions. This inconsistency among existing findings as a result of investigating individual cities imposes uncertainty regarding the application of results to urban land cover planning and management. Accordingly, a consistent global investigation with a high spatial resolution is needed to identify the underlying factors controlling intra urban temperature variability across different cities.

Analyses on Ahmadabad city^29^ in India, Berlin^30^ in Germany, and Baltimore^31^ in the U.S. suggested that the effects of the land cover indices on LST are season-dependent. This has led to the conclusion that the relationship between land cover parameters and LST is suitable mainly for the summer season^31^. However, the validity of this general concept has not been confirmed yet and it may depend on the climate of the city. A comparison study to evaluate the relation between LST and different land cover indices in London, UK and Baghdad, Iraq^32^ found the effects of land cover on LST depended on the local background climate. Converting bare land to built-up areas was found to increase LST in a temperate city such as London, while this land cover conversion could act as a heat mitigation strategy in an arid city like Baghdad. But it is not possible to assess the effectiveness of heat mitigation strategies through studies on a few selected cities as there is not enough basis for generalisation. To address these global issues, a more uniform approach and comprehensive perspective are needed to place current findings in a geographical context and transfer knowledge across climatic zones.

**Figure 1:**
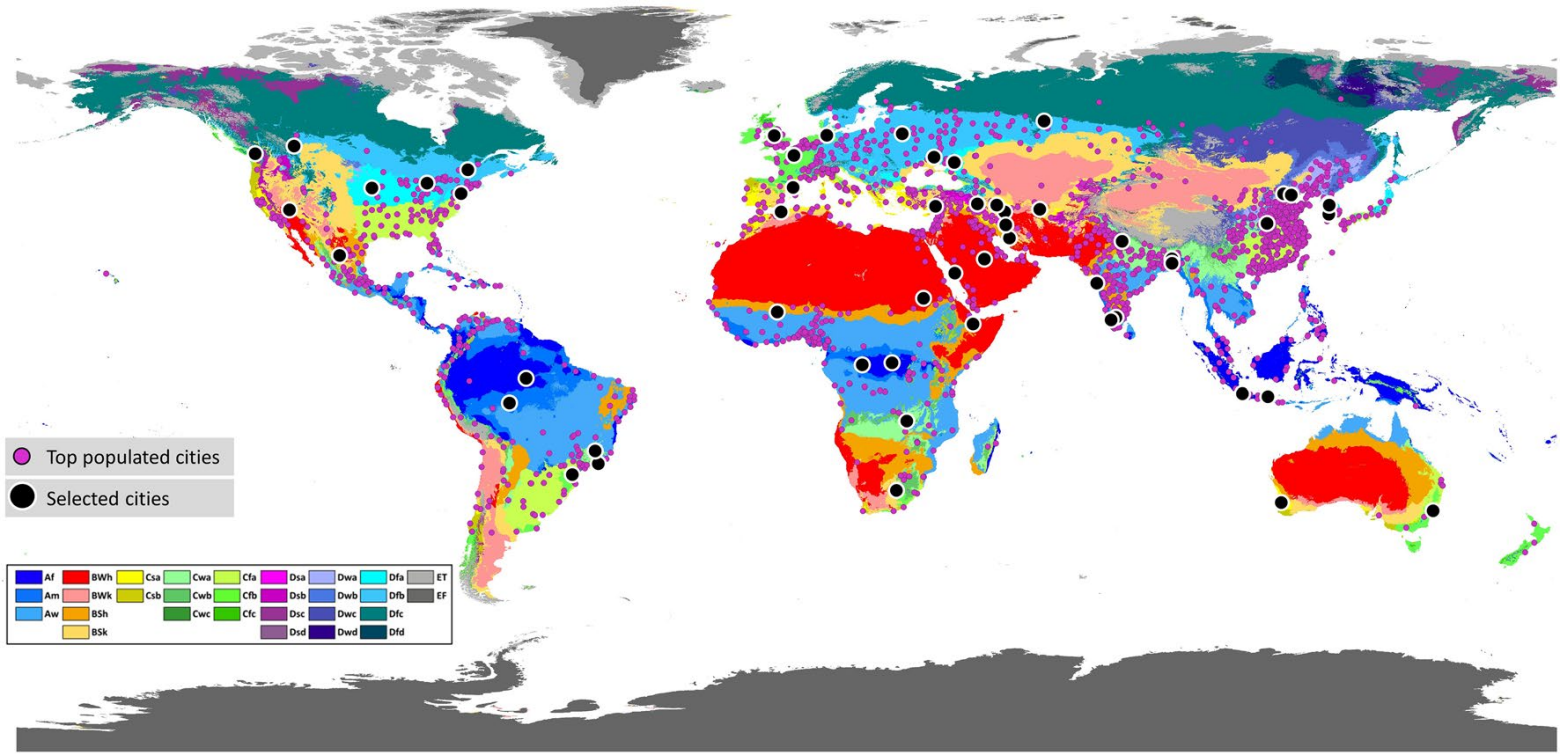
The distribution of the most populated cities and the selected cities for this study on the world map of Köppen–Geiger climate classification. Purple and black symbols depict the top populated cities around the world and the selected cities for this study, respectively (see “Methods” for the details on how to select cities). Cities are grouped by Köppen–Geiger climate classes: tropical, arid, temperate, and cold^33^. The legend in the lower left-hand corner indicates Köppen–Geiger climate subclasses. Cities were located on the map using ArcGIS 10.8 software (www.esri.com/software/arcgis).

Accordingly, we aim to advance the understanding of urban surface characteristics and their impact on intra-urban surface temperature variations using high-resolution remote sensing data. This is the first global study that uses Landsat imagery to investigate surface urban heat and its driving factors at a city scale. We selected 54 cities among the top populated cities around the world (Fig. 1) and explored land cover and surface temperature characteristics for cities located in each of the main climate zones. Cities were categorised based on the updated Köppen–Geiger climate classification system^33^. We find that land surface characteristics and their thermal response are distinctly different across various climatic zones. We also used a Gradient Boosting (GB) regressor to determine the controlling factors of intra-urban surface temperature variability during warm and cold periods in each climate group. Our findings can help improve understanding of how land surface characteristics interact with the spatial structure of surface urban heat patterns. Moreover, results from this study can provide new insight and guideline for quantitatively investigating surface urban heat and land cover properties which help assess the most appropriate strategies to alleviate the adverse effects of urban heat at global and local scales.

## Results

### Land surface characteristics in different climate classes

**Figure 2:**
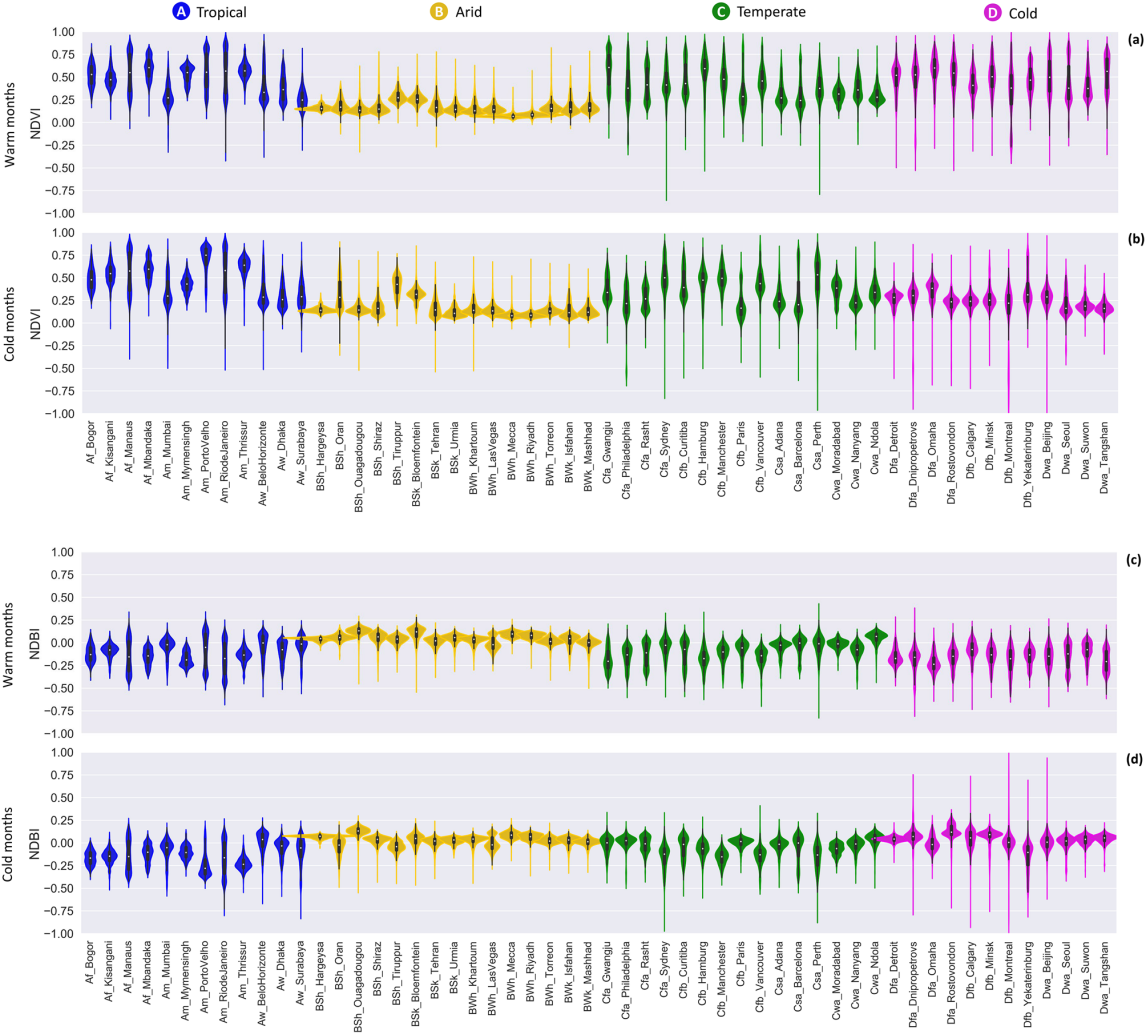
Range and distribution of land cover variable values for 54 cities. Coloured areas represent the distribution of the values. White dots within the violin plots depict the median values. (**a**) NDVI in warm months. (**b**) NDVI in cold months. (**c**) NDBI in warm months. (**d**) NDBI in cold months. Violin plots for NDWI and NDBaI are provided in Supplementary Fig. S2.

As determinants of LST, built-up areas, vegetation, soil, and water were studied in each selected city using four spectral indices (NDBI, NDVI, NDBaI, and NDWI) retrieved from Landsat 8 imagery. To understand how land cover differs across different climate classes, variability in multiple land cover indices is illustrated via violin plots during warm and cold months. NDVI, NDBI (Fig. 2), and NDWI (Supplementary Fig. S2) show similar variability with the change of season and climate zone, while NDBaI (Supplementary Fig. S2) shows a very different pattern. For the first three indices, the index value ranges are smaller in arid cities than other climate classes, resulting in different shapes of distribution. Land cover values in arid cities are highly concentrated around the median, whereas temperate and cold cities have a more elongated distribution, without a distinct peak, especially in cold climates. This is likely attributed to the heterogeneity of the cities; arid cities are more spatially homogeneous in comparison with other climates. In arid cities, most land use consists of built-up surfaces, and the portion of vegetation cover and water bodies is limited. In contrast, in tropical, temperate, and cold climates, cities are more heterogeneous and have more vegetation and water cover even in built-up areas.

The range for the land cover variables does not show a strong seasonal variation in tropical, arid, and temperate climates, while in cold-climate cities, the range of values is season-dependent. In cold climates, the violin plots present a narrow elongated distribution in warm months but a more concentrated distribution during cold months. In this climate class, NDVI values range from 0.2 to 0.9 (average median of 0.49) in summer, whereas during winter, NDVI values are between 0 and 0.5 (average median value of 0.27). This indicates that there is much less actively photosynthesising vegetation during this period. Shedding of leaves from deciduous trees in winter also causes more visibility of other land covers to the satellite, reducing the range and increasing the median values for NDBI (median in summer: − 0.15, in winter: 0.02) and NDWI (median in summer: − 0.50, in winter: − 0.33) in cold cities (see “Methods” for the ranges of indices value for vegetation cover in Table 1). Similarly, NDBaI has a significantly smaller range in winter compared to summer in this climate zone (Supplementary Fig. S2). Receiving less solar radiation during winter may also increase surface moisture, resulting in a lower NDBaI in cold cities. In addition, the range of NDBI in almost all cities is smaller than the range in NDVI and NDWI, which may indicate less sensitivity of NDBI to the variation of surface characteristics in cities compared to NDVI and NDWI.

**Figure 3:**
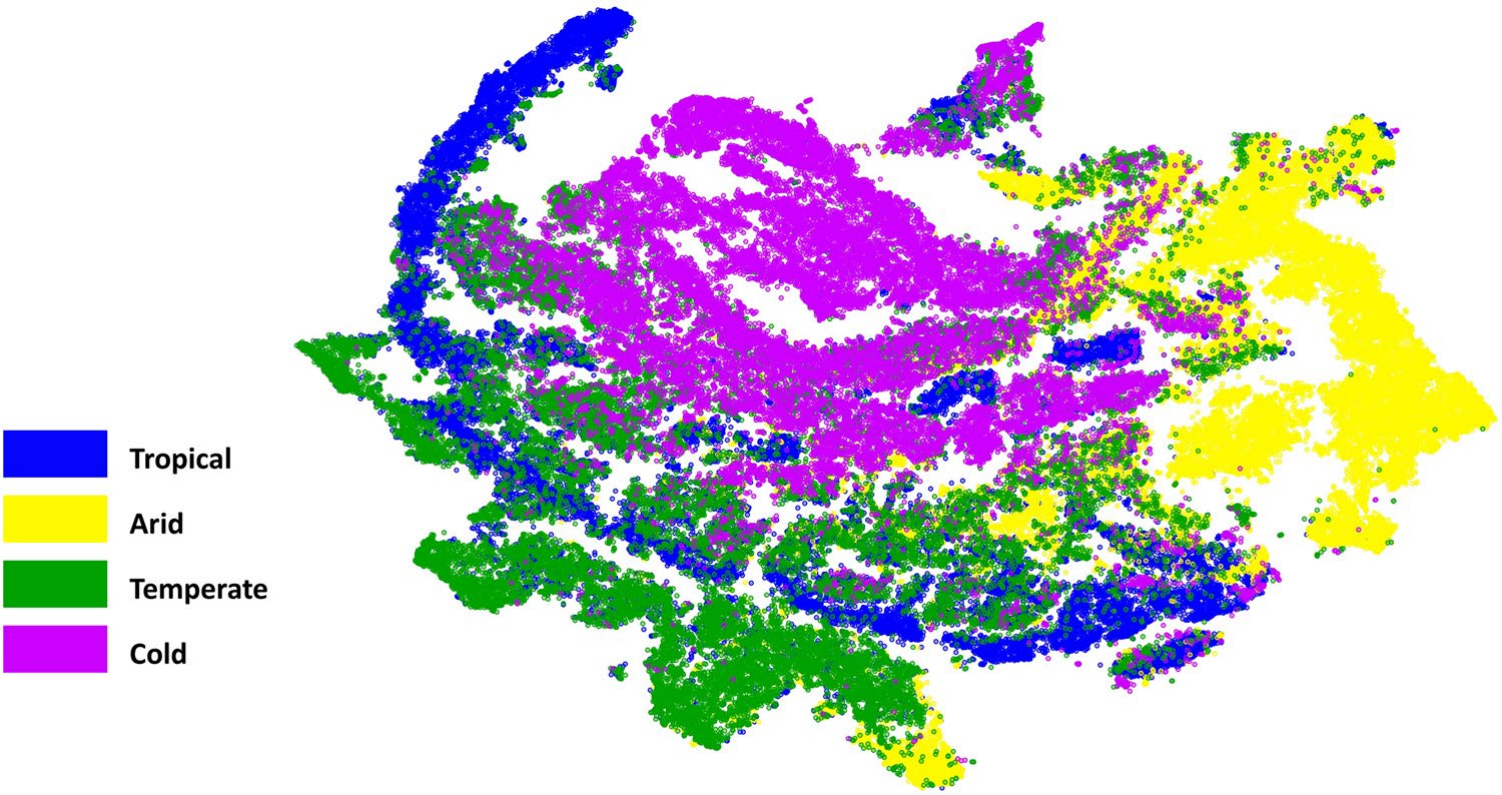
Distinct climate classes based on their land surface characteristics, visualised using t-SNE. Grid cells were arranged in 2 dimensions based on similarity of their land cover data by t-SNE. Each data point represents a single grid cell coloured by climate class. This figure was generated using the open-source python package scikit-learn (v.1.0.2)^35^.

To further identify the difference in surface characteristics between climatic zones, the t-distributed Stochastic Neighbor Embedding (t-SNE)^34^ algorithm was used here. By mapping the land cover data from the original 6-dimensional space (NDVI, NDBI, and NDWI during warm and cold months) to a 2-dimensional space, t-SNE allows visualisation of hidden clusters of the data. Clusters are more pronounced with these land cover parameters and do not depend on NDBaI. Using this technique, we could reproduce the described climatic zones and show which climate classes are distinct, more diffuse, or might share similarities with other groups. The t-SNE plot (Fig. 3) shows that urban surface characteristics in arid cities are distinct from other climate classes as the majority of data points formed a separate cluster. The significant difference for arid cities can also be confirmed by Fig. 2, in which individual land cover variables were characterised during warm and cold months using violin plots. While the overall unique pattern of the other climate classes is also distinguishable, there are overlaps in some parts. Temperate climate overlaps with tropical and cold climates, showing partial similarities between these climate classes in surface characteristics, whereas cold and tropical climates form two separate clusters. One of the reasons for this distinct behaviour between cold and tropical climates may be due to the seasonal variations in these climate classes. Cold climate indicates the largest seasonal variability in land cover, while the lowest can be observed in tropical and arid climates (Fig. 2). These results emphasise that land surface characteristics are distinctly different in different climatic zones.

### Variability of land surface temperature in different climate classes

**Figure 4:**
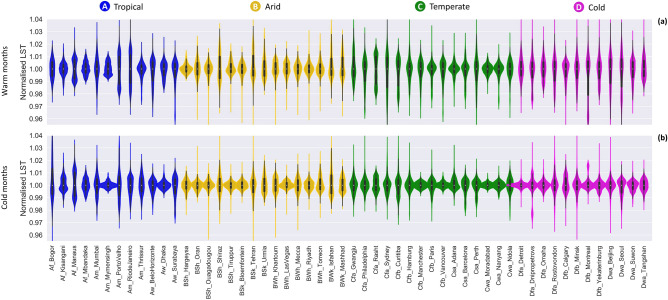
Violin plot illustrating the range and distribution of LST values for 54 cities. Coloured areas represent the distribution of the values. White dots within the violin plots depict the median values. (**a**) Normalised LST in warm months. (**b**) Normalised LST in cold months.

Here, we assess the variability of land surface temperature in different climates and with different distributions of land covers. To compare LST variations across different seasons and climatic zones, the LST value of each grid cell was normalised using the median LST value in each city. Figure 4 shows a larger range for LST during warm months in all cold climate cities and those temperate cities in subclasses Cfa and Cfb (humid subtropical and temperate oceanic climates). This may be due to the fact that cities in cold and temperate (Cfa and Cfb) climates have more heterogeneous land cover distributions than other climate classes. A similar pattern can be seen in Fig. 2, which displays the range and distribution of land cover variable values in different climate groups. Except for the tropical climate, LST values show seasonal variation in all cities. The ranges of LST values are smaller and the width of density curves are increased during winter, especially in cold climate cities. The largest seasonal variability in cold cities may be attributed to the distribution of the deciduous trees in the cities and the variability in climatic conditions between seasons. In tropical cities, the ranges of LST values remained relatively constant during warm and cold months. This is likely because these regions are close to the equator and experience the smallest yearly variation in solar radiation.

### Bivariate associations between LST and land cover variables across different climatic classes

Here, the correlations between land cover variables and LST are evaluated in different seasons and background climates. We used scatterplots to explore the linear association between land cover variables and LST and their slope in each city. First, the NDVI-LST correlation can provide information on surface moisture conditions. The slope of this relationship is related to the evapotranspiration rate of the surface^36^. The main controlling factors of surface evapotranspiration are surface moisture, radiation, and the presence of active vegetation^37^. In most cities, the effects of NDVI and NDBI on LST show similar linear patterns but with different directions (see Supplementary Figs. S3 and S4), whereas NDBaI-LST relationships are mostly nonlinear (see Supplementary Fig. S5). This may be due to the fact that the effects of soil on LST cannot be the same for the entire range of soil. NDVI shows a negative correlation with LST, while NDBI illustrates a positive relationship with LST, which indicates the cooling effects of vegetation cover and the warming impact of built-up areas in cities. Moreover, due to less vegetation cover and surface moisture, slopes of NDVI-LST relationships are steeper in arid cities. This is consistent with the results of previous studies, reporting that the slope of NDVI-LST correlation is steeper in areas with less soil moisture and vegetation amount^38,39^.

**Figure 5:**
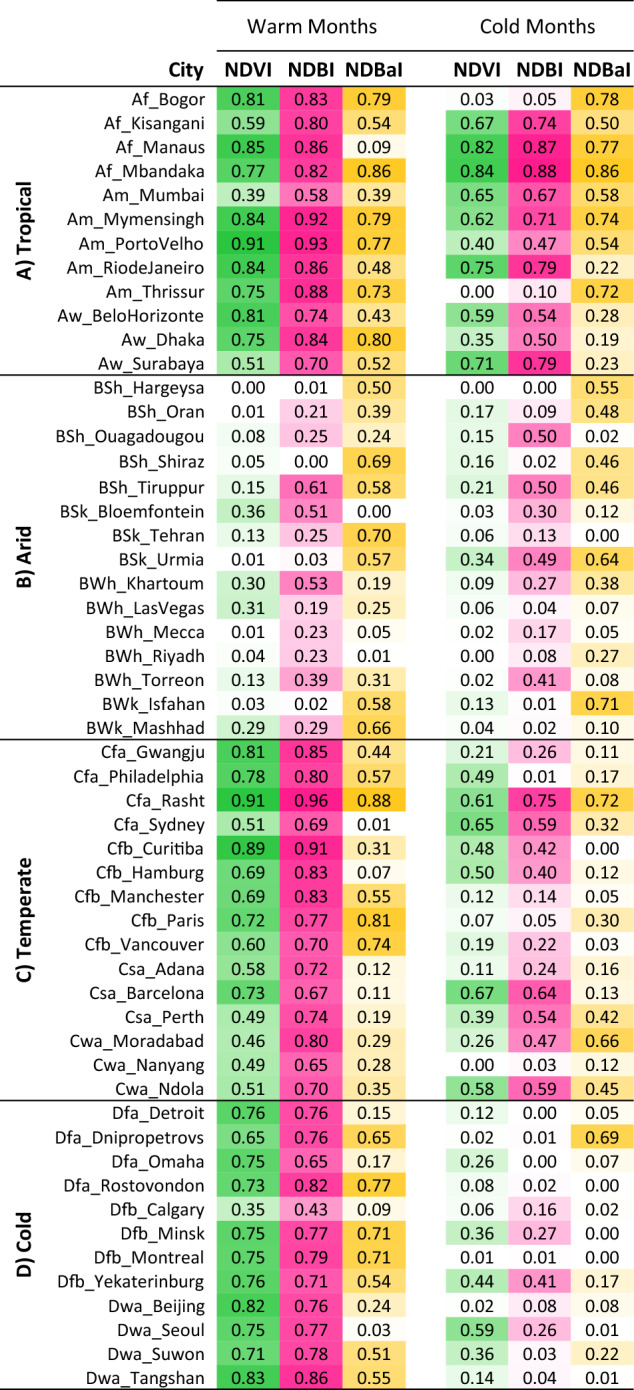
The estimated coefficients of determination (R^2^) between land cover variables (NDVI, NDBI, NDBaI) and LST for 54 selected cities during warm and cold months. The colour gradient is for ease of comparison of R^2^ values. Darker colours represent stronger relationships, while lighter colours indicate weaker correlations.

To better understand the effects of background climate and seasonal variability on the strength of bivariate associations between the land cover variables and LST, a correlation matrix was developed for all 54 cities. Previous studies have reported a stronger correlation between land cover variables (such as NDVI and percent of imperviousness) and LST during summer than winter, suggesting that land surfaces have greater impacts on LST during summer^31,40^. However, our results show that the background climate of a city controls the relationship between land cover variables and LST (Fig. 5). In warm months, LST is highly correlated with NDVI and NDBI in tropical, temperate, and cold climates, however, little correlation is seen in arid cities due to evapotranspiration and water stress.

The relationship between the land cover variables and LST in arid and tropical climates does not show a significant seasonal variation. This can be explained by the fact that the determinants of evapotranspiration (radiation, surface moisture, and vegetation status) remain relatively constant during warm and cold months, and the ranges of LST (Fig. 4) and NDVI (Fig. 2) in warm months are approximately similar to the range in cold months in these climatic zones. Conversely, in cold and temperate climates, the effects of land cover variables on LST are season dependent. The cooling effect of vegetation on LST is much stronger during warm months than cold periods in some temperate cities and all cold cities (Fig. 5). The greater seasonal variability in cold climates may be attributed to the distribution of the deciduous vegetation within the cities and the seasonal variability in climatic conditions.

The NDVI-LST relationship is slightly stronger than the NDBI-LST correlation in most cold cities during winter, while the opposite is mainly observed in other climatic zones. The comparative strength of NDVI-LST and NDBI-LST linear relationships in tropical, temperate, and cold climates is governed mainly by the range of these two land cover variables. A smaller range of NDBI compared to NDVI (Fig. 2) results in a weaker NDBI-LST correlation in most cold cities during winter but a slightly stronger relationship in other cities in both warm and cold periods. The smaller range can cause a steeper slope for NDBI-LST correlations as the range of the dependent variable (LST) is constant. The steeper slope means higher R^2^ if the standard deviation (Std. Dev.) does not change significantly. This explains the slightly stronger NDBI-LST relationship compared to the NDVI-LST correlation in most temperate and tropical cities during warm and cold months and most cold cities during the warm months. However, LST has very small variations in cold cities during the cold period (Fig. 4), and if the dependent variable has a very small range, the independent variable with larger variation (NDVI has a bigger range than NDBI) can have a higher R^2^, which explains the slightly stronger effects of NDVI on LST compared to NDBI in most cold cities during winter.

### Explanatory power of land cover and terrain variables on LST

**Figure 6:**
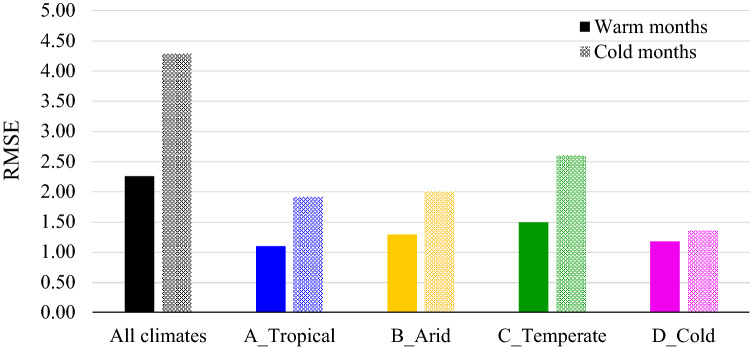
Explanatory potential of all variables on LST, measured by RMSE. A comparison of the explanation rate during warm and cold months before and after classifying climate.

To explain the variance in LST, we investigate NDVI, NDBI, and NDBaI, alongside albedo, distance from the coast, and elevation, based on knowledge derived from previous studies of important determinants of SUHI^13,41^. Using the combination of these variables in a GB regression model, we could better explain the variance in LST during warm months (RMSE = 2.26 °C, adjusted R^2^ = 0.90) than the cold period (RMSE = 4.29 °C, adjusted R^2^ = 0.78; Supplementary Table S2). This shows the stronger predictive capacity of the explanatory variables during warm months. Figure 6 illustrates that classifying climate substantially enhances the explanatory power of land cover and terrain variables on LST in both warm and cold periods. These variables could slightly better predict LST in tropical and cold climates compared to arid and temperate climates. The prediction of LST in temperate climate cities had the largest error in both warm (RMSE = 1.49 °C, adjusted R^2^ = 0.88) and cold periods (RMSE = 2.61 °C, adjusted R^2^ = 0.80, Supplementary Table S3). The pattern is similar to the results in Fig. 5; cities in temperate climates tend to have the highest intra-group variation in the relationship between land cover variables and LST, especially during cold months, while cities in tropical and cold climates have the lowest. This may be attributed to the greater spatial dispersion in temperate climates compared to other climatic zones.

### Contributing factors to LST variability

To understand which variables contribute the most to explaining the variance in LST, we extracted the importance of features using the GB model. This decision tree-based algorithm showed superior performance compared with other machine learning models here, consistent with a number of studies^42,43^. It creates a series of decision trees—where each tree attempts to minimize the errors made by the previously trained tree—to achieve optimization slowly but steadily. GB improves the accuracy of the final regression results by using the strengths of regression trees and boosting^34^.

**Figure 7:**
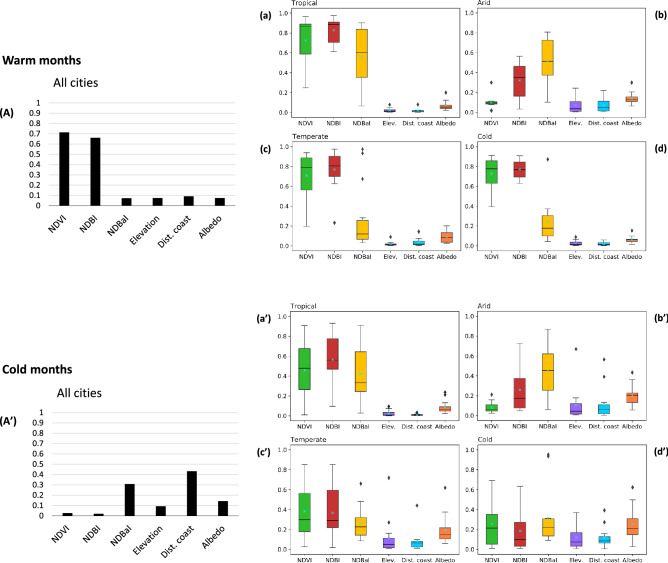
Determinants of LST variability. Predictor feature importance scores extracted from trained GB model explaining the variance in LST during warm months (**A** all cities before classifying climate, **a** tropical cities, **b** arid cities, **c** temperate cities, **d** cold cities) and cold months (**A’** all cities before classifying climate, **a’** tropical cities, **b’** arid cities, **c’** temperate cities, **d’** cold cities).

When combining all cities (without considering their climate class), NDVI and NDBI have the largest contribution relative to the other factors in LST variation in summer (Fig. 7A). However, the contribution of NDVI and NDBI decreases in winter, and the dominant explanatory factors change to distance from the coast and NDBaI (Fig. 7A’). The moderating influence of the ocean in winter is less pronounced when this analysis is done separately for cities in each climate class. It brings out the importance of the other actual land cover variables in LST variability during winter. Figure 7 also indicates the extracted feature importance for the same explanatory data but after classifying climate. The dominant factors of LST variability in arid and tropical climates do not show a significant seasonal variation. In contrast, in cold and temperate climates, the controlling factors of LST are season-dependent, especially for cold climates. In tropical, temperate, and cold climates, NDBI has the most significant contribution to the variability in LST during warm periods followed by NDVI, while NDBaI is the most important factor in arid climates, followed by NDBI and albedo. In temperate and cold climates, NDVI and NDBI are less influential in explaining LST variations during cold months, whereas NDBaI, Albedo, and terrain variables (distance from the coast and elevation) are slightly more important in this period compared to warm months. In tropical climates, NDBaI shows large variability (high Std. Dev.), which means soil may be important in some but not all tropical cities. This is likely because there might be some other regional factors (such as soil moisture and average precipitation) influencing soil characteristics and its thermal response in this climate class. In general, NDBI can be the best predictor of LST with low Std. Dev. and relatively consistent explanatory power across different climatic zones (except arid), accounting for the majority of the LST variation during summer. With less importance, it can also be a useful complement to NDBaI for surface urban heat modelling in arid cities.

## Discussion

This study implements the first global scale data-driven assessment of urban surface characteristics and how they influence the intra-urban surface temperature variability. Our results quantify these effects across different climatic zones, confirming the need to consider the background climate of the city in order to assess surface urban heat. In particular, this assessment indicates that urban land surface characteristics and their thermal response are distinctly different in diverse background climates, with the largest difference for arid cities. Previous studies reported that arid and semi-arid areas have different spectral characteristics and thermal behaviour relative to other climatic regions^44^. However, our global climate-zone-based assessment is the first study quantifying how arid cities differ from cities in other climatic zones.

Past regional and global studies have investigated SUHI intensity in different cities using MODIS imagery^20,22^. These studies measured the SUHI intensity across a city by calculating the surface temperature difference between urban areas and surrounding suburban areas. This led to the conclusion that the intensity of SUHI is weaker in arid cities^20,22^. However, it is known that arid cities often experience higher levels of urban heating and subsequent increases in building energy consumption and thermal discomfort, motivating closer attention to intra-urban variability in absolute surface temperature as opposed to urban–rural comparisons.

Through characterising land cover variables (NDVI, NDBI, NDBaI, Albedo, and NDWI), we demonstrate that focusing on SUHI in arid cities can indeed yield misleading results. Land cover variables have a smaller range in arid cities (Fig. 2), indicating less variation and more similarity in physical, thermal, and radiative properties of the land covers (such as surface roughness, albedo, and aerodynamic resistance) due to low vegetation and water cover within built and surrounding rural areas. This can explain the lower urban-suburban surface temperature difference (i.e., weaker SUHI intensity) extracted for dry cities in previous studies. More importantly, spatial maps of LST in arid cities (Supplementary Fig. S1) demonstrate lower LST in urban areas compared to non-urban surroundings. Accordingly, we demonstrate the critical importance of evaluating intra-urban temperature variability, rather than simply measuring the temperature difference between urban and surrounding areas, for assessing urban overheating and informing corresponding mitigation strategies.

Our results also show that seasonal differences of intra-urban surface temperature variability are largely controlled by background climate. LST values have larger variations during warm months than in cold periods (Fig. 4). This seasonal variation of LST in different cities may be explained by the intensity of total solar radiation and the evaporative cooling effects of the vegetation. Seasonal LST variation is more pronounced in cold, as well as humid temperate cities, as there is more seasonal greening contrast between urban and surrounding areas in these regions. On the other hand, as vegetation remains relatively active through the whole year in tropical cities, the least seasonality in LST variation is observed in these cities.

Results from this study also illustrate that the relationships between land cover variables and LST are different across different cities (Fig. 5), and the strength of these correlations is closely related to the climatic and seasonal factors. As the variations of land cover variables and LST (Figs. 2, 4, respectively) vary with climatic regions and seasonal variability, the correlation between land cover indices and LST are also likely connected with these two.

Vegetation modulates the contribution of latent and sensible heat fluxes to the surface energy balance and generates a cooling effect on land surface temperature via transpiration^22^. The correlation matrix (Fig. 5) and the scatterplots (Supplementary Fig. S3) show the cooling mechanism as NDVI (as the indicator of vegetation activity) is negatively and strongly correlated with LST across most cities (except cities in arid climates) during warm months. The weaker relationship in dry cities is due to evapotranspiration and water stress. In this climate, the heating and cooling effects of the surface are largely controlled by moisture availability; cities act as a source region for sensible heat in the absence of urban irrigation. Vegetation cover can have a significant cooling effect in arid regions only if urban irrigation is supplemented^45,46^.

Through characterising NDVI during warm and cold months (Fig. 2a,b, respectively), the seasonal variation of vegetation can be clearly seen in cold cities. Cold climates tend to have a higher latitude than other climatic zones. Thus, land surfaces receive less solar radiation due to lower solar altitude, larger sun zenith angle, and shorter duration of sunlight in winter. In addition, the evapotranspiration rate in the canopy layer is significantly reduced in this season due to shedding leaves from deciduous trees, which reduces the trees' capability to regulate LST^31^. The lower temperature in winter also significantly decreases evapotranspiration in vegetated areas and increases surface moisture, reducing the strength of the correlation between NDVI and LST^36,37,47^. These explain a much weaker correlation between NDVI and LST in winter for cold cities. A relatively large seasonal variability in the correlation between NDVI and LST was also found in the Twin Cities Metropolitan Area of Minnesota, located in cold climate^48^.

Compared to suburban areas, urban areas have lower surface albedo and emissivity, larger heat capacity, and higher heat conductivity which increase surface heat storage during the day^49,50^. Further, the intensity of radiation trapping is increased with increasing the density of built-up areas^51^. High values of NDBI signify areas with densely built urban environments^52^. Significantly positive relationships between NDBI and LST were observed in around 76% of cities during summer and around 35% of cities during winter (Fig. 5 and Supplementary Fig. S4). Weak NDBI-LST correlations in cold cities during winter may be attributed to the lower solar altitude which causes tall buildings to more easily form shadows in this climate class, decreasing the direct shortwave radiation in winter^17^. However, the observed pattern for NDBI is very similar to NDVI for most cities which shows the thermal effect of vegetation changes was negatively coupled with the impact of spatial variation of built-up area in cities.

NDBaI shows distinct behaviour while comparing its range across different cities (Supplementary Fig. S2) and its relationship with LST (Fig. 5). This index can signify the distribution of soil in urban areas^53^. Although previous studies reported that the difference in soil characteristics can explain the effects of climate^45,54^, NDBaI in this study does not exhibit clear patterns based on the background climate. This finding highlights the importance of considering variables that better characterise the nature of soil in different climates in future surface urban heat studies.

Given the uncertainty of the individual input variables, we explored the combination of these land cover parameters along with albedo and terrain variables (elevation and distance from the ocean) to explain the variance in surface temperature variability. Overall, the machine learning model used for the analysis confirmed that the parameters integrated in this study can collectively well explain the variance in LST.

We find the strong contribution of background climate to the explanatory power of land cover and terrain variables on LST during different seasons. For instance, NDVI is a poor predictor of LST in arid cities, while it can significantly contribute to surface temperature in tropical, temperate, and cold climates. LST variation can be better explained during warm months than the cold period (lower RMSE and higher adjusted R^2^ values for summer than winter, Fig. 6 and Supplementary Table S2), indicating compounding mechanisms contributing to surface temperature variability during winter. This result is different from the findings of a study investigating diurnal and seasonal SUHI intensity in China’s 32 major cities using MODIS products^20^. This is likely due to this regional study investigating just the urban-suburban LST and land cover differences rather than intra-urban variability. However, our findings are consistent with the simulation results of seasonal LST in Dhaka Metropolitan area using artificial neural networks–cellular automata (ANN-CA) model^55^. While there is no significant season dependency in tropical and arid climates, the dominant factors of LST variability in temperate and cold climates are season-dependent (Fig. 7). Similar results with vegetation-related factors have been found in Baltimore, Maryland, which is a temperate region^31^.

We also find that NDVI and NDBI are important contributors to LST in tropical, temperate, and cold climates during summer. Therefore, urban greening and controlling the expansion of built-up areas remain important strategies for the goal of mitigating the effects of surface urban heat in tropical, temperate, and cold climates. Nonetheless, in arid cities, NDBaI is the dominant factor controlling LST variability (followed by NDBI), highlighting the important influence of soil and built-up areas on the surface urban heat in dry cities. However, we do note the spectral characteristics of the urban area and bare soil are relatively similar and may result in sensitivity when classifying these two land covers while using NDBaI in arid cities. In arid and semi-arid climates, greening is constrained by water limitations. Therefore, an alternative heat mitigation option may involve innovative pervious surface covering, controlling building height and spacing, and depending on water availability, growing a ring of irrigated crops in the surroundings to improve the thermal environment. Compared with a previous study on summer urban heat island over European cities^41^, the explanatory rate of albedo, distance from the coast, and elevation on LST during summer is low in tropical, temperate, and cold climates. These factors are slightly more important in arid cities (during summer and winter) and temperate and cold cities during winter. A slightly more evident effect of albedo on LST variation during winter resulted from vegetation defoliation and/or antecedent ice and snow coverage in this season.

The detailed spatial information presented by this study provides a fundamental understanding of urban surface characteristics and their thermal response across diverse climatic zones. We expect that the global assessment presented here could further serve as a theoretical basis for assessing surface urban overheating and the implementation of land cover planning to improve the urban thermal environment. However, some limitations in this study need to be mentioned. As Landsat satellite provides information on daytime surface temperature, the current analysis was conducted only for daytime, while diurnal variability needs to be taken into account for a more in-depth investigation. In addition, our study is limited to spatial variability, making a prediction of how surface urban heat will respond under climate change scenarios would require a temporal analysis as well. Furthermore, future works could explore other land cover products, as they become increasingly available, that characterise different urban land cover types in order to provide a mechanism for quantitatively assessing thermal impacts of urban land covers in different climatic regions.

## Methods

### Framework and analysis process

**Figure 8:**
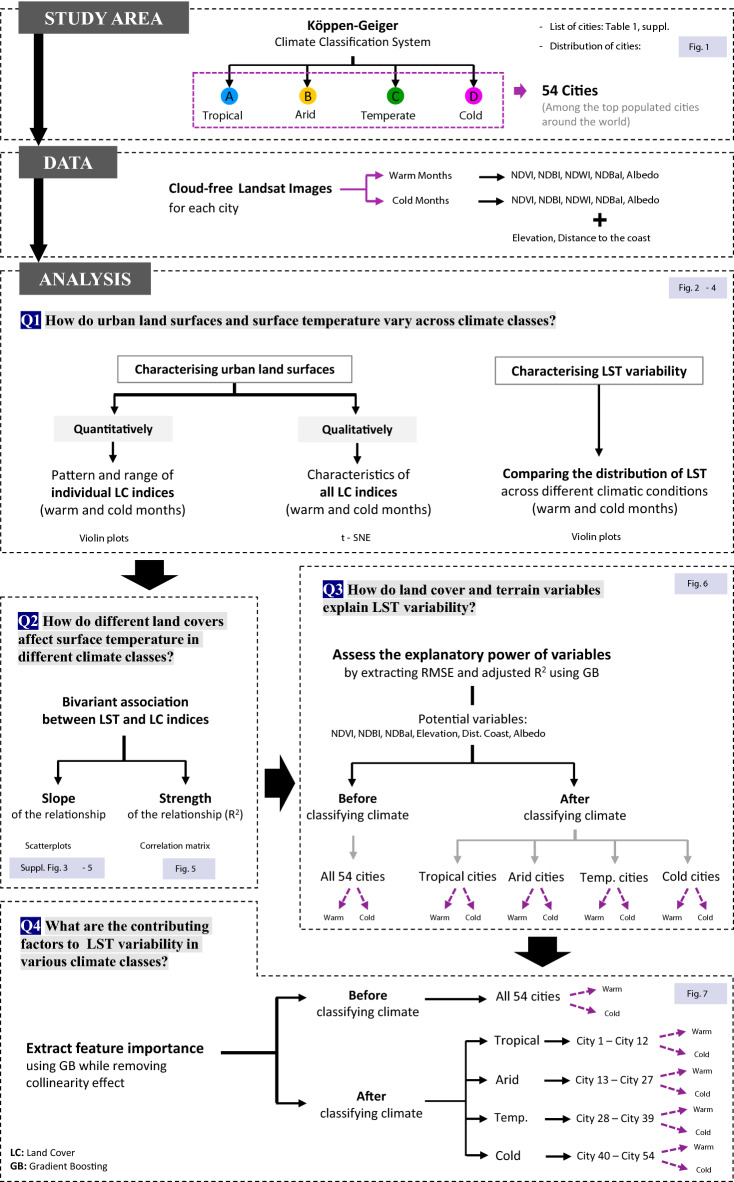
The framework of the study.

To explore the effects of background climate on urban land surfaces and their effects on surface urban heat, we first characterised land cover (quantitatively and qualitatively) and LST variability in different climatic conditions. The bivariate association between LST and land cover indices was also investigated across diverse climatic zones during warm and cold months. To obtain in-depth analyses of the relationship between land cover variables and LST, we applied machine learning methods. A GB regression model was used to explain the variance in LST before and after classifying climate. Finally, we extracted feature importance to understand which variables contribute the most to explaining the variance in LST. A flowchart has been added to describe the framework of this study as a whole (Fig. 8).

### Selecting urban areas in representative climate classes

**Figure 9:**
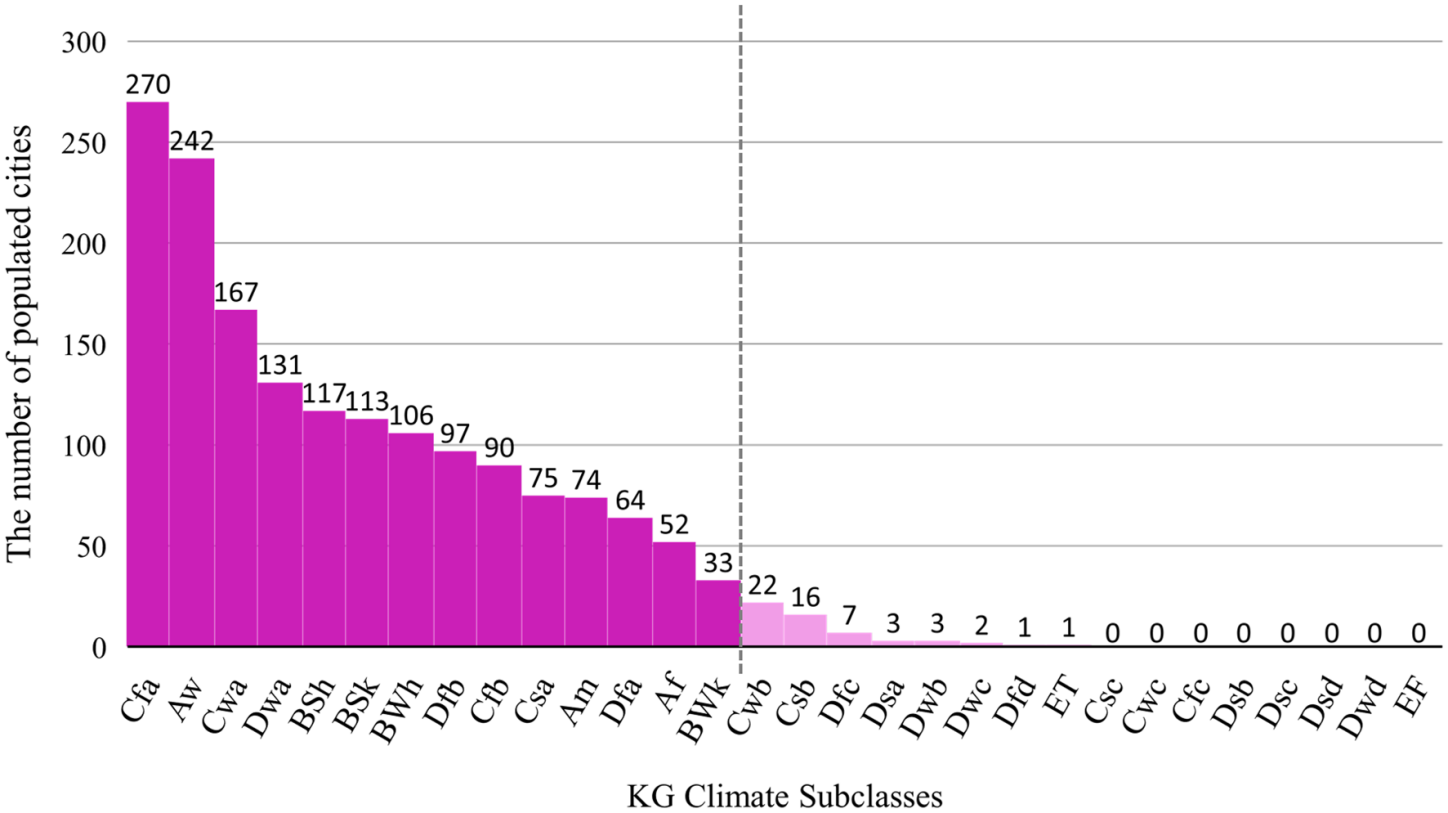
The number of the top populated cities in each Köppen–Geiger climate subclass.

We selected 54 cities among the most populated cities around the world^56^. To ensure that representative climate classes are included, we mapped the distribution of the most populated cities (with more than 300,000 inhabitants) worldwide against Köppen–Geiger climate subclasses (Fig. 9). This exercise determined that only 22 climate subclasses had one of the top populated cities situated within. Accordingly, only climate subclasses with more than 30 cities were studied in this analysis and each climate subclass is represented by 3–5 cities considering the availability of cloud-free satellite images during the warm and cold months. Keeping the consistency between different cities, we drew the boundary of each city based on Google map urban boundaries. Where local literature was available, these boundaries were modified to align with the previous research. The list of cities, their representative climate class, and the distribution of the selected cities on the Köppen–Geiger climate classification map are shown in Table S1 (Supplementary file) and Fig. 1, respectively.

### Selecting warm and cold months in individual cities

The seasons in the southern hemisphere are the opposite of those in the northern hemisphere. Furthermore, not all climate classes experience distinct summer and winter seasons. For instance, tropical cities (12 selected in this study) are more likely to experience wet and dry seasons instead. Considering these factors, we selected the periods representative of cool and warm months based on the climatology of each city (and not months of the year) and the satellite images were categorised into warm and cold months accordingly.

### LST, land cover, and terrain data

Landsat 8 satellite imagery is used for determining LST and land cover indices. For each city, we selected 2–4 cloud-free Landsat 8 images in each warm and cold season (captured in a year between 2017 and 2020) via the United States Geological Survey (USGS), Earth-explorer website (earthexplorer.usgs.gov). The Landsat images are collected approximately every 16 days at 10:00 am (± 15 min) mean local time and thus provide information on daytime surface temperature. For most cities, we selected the Landsat scenes that cover the whole city. However, where Landsat scenes were not available for a whole city, two scenes captured simultaneously were used. LST and land cover indices were computed and extracted using Google Earth Engine (GEE) cloud-computing platform^57^. Figure S1 (Supplementary) shows the extracted LST maps for 54 selected cities during the warm month. For calculating LST, we used the Statistical Mono-Window (SMW) algorithm developed by the Climate Monitoring Satellite Application Facility (CM-SAF) to derive LST records from Meteosat First Generation (MFG) and Second Generation (MSG) series of satellites^58^. It employs an empirical relation between top of atmosphere (TOA) brightness temperatures and LST in a single thermal infrared (TIR) channel. The model is based on a linearisation of the radiative transfer equation with an explicit reliance on surface emissivity which was extracted from the Advanced Spaceborne Thermal Emission and Reflection Radiometer Global Emissivity Database (ASTER GEDv3) with an NDVI-based correction for vegetation changes.

1 $$LST = A_{i} \times T_{b} /\varepsilon + B_{i} \times { 1}/\varepsilon + C_{i}$$

where *T*_*b*_ refers to the TOA brightness temperature in the TIR channel and ε refers to the surface emissivity of the channel. *A*_*i*_, *B*_*i*_ and *C*_*i*_ are the model coefficients calibrated for the classes with different total column water vapor (TCWV) values and view zenith angle. This algorithm is implemented by Ref.^59^ in the GEE.

Vegetation, water, soil, and built-up areas are represented using four spectral indices, Normalised Difference Vegetation Index (NDVI)^60^, Normalised Difference Water Index (NDWI)^61^, Normalised Difference Bareness Index (NDBaI)^53^, and Normalised Difference Built-up Index (NDBI)^62^. The indices range from − 1 to + 1, and different ranges are commonly used to differentiate the prevalence of various land covers (Table 1).

**Table 1:** The ranges of indices indicating the different land cover types^61,63^.

	NDVI	NDBI	NDBaI	NDWI
Vegetation	> 0.2	< 0	< -0.25	< 0
Built-up	< 0.2	0.1–0.3	< -0.2	< 0
Bare soil	< 0.2	> 0.25	> -0.1	< 0
Water	< 0	< 0	< -0.65	> 0

Previous studies have shown that there is an optimal spatial scale to explore the effects of land cover patterns on LST^64,65^, and this can be an integer multiple of the satellite image's pixel size^66^. As the spatial resolution of Landsat thermal and multispectral bands are 100 m and 30 m, respectively, the grid cell size of 300 m × 300 m was chosen to investigate urban land surface characteristics and the effects of land cover variables on LST in different climatic zones.

To analyse the influence of different land cover variables on LST, water bodies were excluded as previous studies note the distinct thermal behaviour of water pixels. LST is negatively correlated with vegetation indices in non-water areas, whereas they are positively correlated in water body sites^67,68^. We extracted three water indices: NDWI, Modified Normalised Difference Water Index (MNDWI)^69^, and Automated Water Extraction Index (AWEI)^70^ and removed the positive values which represent water pixels.

To determine the controlling factors of LST, in addition to the primary predictor variables (NDVI, NDBI, and NDBaI), we collected three other predictors: albedo, elevation, and distance to the ocean. Albedo was computed by the algorithm developed by Ref.^71^ and normalised by Ref.^72^ using Landsat images for each city. Elevation was calculated from NASADEM dataset at 30-m resolution^73^. We also extracted the distance to the ocean using the layer provided by NASA at 0.01-degree resolution (https://oceancolor.gsfc.nasa.gov/docs/distfromcoast/).

### t-SNE

We applied t-SNE method to better characterise the land cover indices and visually analyse the similarity of urban surface characteristics in different climate classes. t-SNE is a statistical clustering method for visualising high-dimensional data and identifying similarities between data points in such a way that dimensionality can be reduced while the local structure is reserved^34^. The t-SNE method produces a 2D visualisation where similar data points in the high dimensional space are represented by a data cluster appearing close to each other. Using this technique, we could map the high dimensional land cover dataset (6D: NDVI, NDBI, and NDWI during warm and cold months) to a 2-dimensional representation. The t-SNE analysis was undertaken using 25,000 randomly selected grid cells for each climate group with 2000 iterations (until reaching a stable configuration) and a perplexity value of 200. This random selection represents approximately 25% of the total dataset. However, the overall observed behaviour for each climate class was relatively distinct regardless of the random selection. Perplexity in t-SNE is a parameter that controls the number of close neighbours each point is attracted to. The ideal value depends on the sample size and different values should be explored^74^. To select an appropriate perplexity, we analysed multiple plots with different perplexity values and found that the results converged to a clear cluster distinction at a perplexity value of 200 and higher. It is also found that the perplexity of 200 produces superior results for a large dataset^75^.

### GB modelling

We deployed machine learning (using the scikit-learn Python package) to obtain in-depth analyses of the effects of land cover variables on LST variation in different background climates during different seasons. GB regression used in this study is a machine learning technique in which decision trees are combined sequentially—a new tree is created at any time step based on the previous performance (boosting method)—to make a robust model that reduces the prediction error^76^. Empowered by the boosting method and shallow trees that avoid overfitting, GB illustrated exemplary performance in various tasks across different disciplines^77,78^. We used GB regression to determine the contribution of the independent variables—NDVI, NDBI, NDBaI, albedo, elevation, and distance to the ocean—on variations of LST as the target variable. In all analyses, the model was trained and validated on 70% of the data, while the rest was withheld for the performance evaluation. We measured the trained model fit and precision by adjusted R^2^ and RMSE (Supplementary Table S2), respectively. The entire process was performed ten times with varying random portions of data as the test and train sets to increase the robustness of the results.

In a GB model, the variable importance is a measure of how much each variable reduces the variance of the model fit. To explore the effects of background climate, we first combined all grid cells without considering their climate class and extracted the importance of individual explanatory variables. Then we classified climate and extracted the importance of each variable for each climate. Before extracting the variable importance, we minimised the effect of potential multicollinearity. Collinearity distorts model estimation when correlation coefficients between explanatory variables are higher than 0.7^79^. As the correlations between the variables vary from city to city, the feature importance was extracted iteratively; for each city in each climate class, different permutations of n variables—n was chosen aiming at maximising the number of predictors for model training—that satisfied the collinearity threshold (R = 0.7) was used to train the GB model. The importance of features was then extracted if the model had the acceptable prediction power on the corresponding test dataset (R ≥ 0.8). The entire process was repeated ten times to eliminate the sensitivity of the framework to random sampling (train/test splitting). Finally, the range of importance scores was plotted for all cities in each climate in the boxplots.

## Supplementary Information

Supplementary Information.

## Data Availability

The raw data for these analyses was gathered from Landsat 8 freely available in GEE and USGS website and the analysed dataset during the current study is available from the corresponding author on reasonable request.
