# Supplementary material for: Background climate modulates the impact of land cover on urban surface temperature
Source: Sci Rep. 2022 Sep 14;12:15433. doi: 10.1038/s41598-022-19431-x (PMC9474840; doi:10.1038/s41598-022-19431-x)
Supplement: Supplementary file 1 — Supplementary Information. [file 41598_2022_19431_MOESM1_ESM.docx]

Supplementary Materials

**Background climate modulates the impact of land cover on urban surface temperature**

Marzie Naserikia^1*^, Melissa A. Hart^1^, Negin Nazarian^1,2,3^, Benjamin Bechtel^4^

^1^ Australian Research Council Centre of Excellence for Climate Extremes, University of New South Wales, Sydney, Australia

^2^ School of Built Environment, University of New South Wales, Sydney, Australia

^3^ City Futures Research Centre, University of New South Wales, Sydney, Australia.

^4^ Department of Geography, Ruhr-University Bochum, Bochum, Germany

^*^ email: m.naserikia@unsw.edu.au

**Table 1** **Selected urban areas and their representative Köppen–Geiger climate class and subclass.**

| **KG class** | **Sub**  **class** | **Definition** | **Selected city** | **KG**  **class** | **Sub**  **class** | **Definition** | **Selected city** |
| --- | --- | --- | --- | --- | --- | --- | --- |
| **A) Tropical** | Af | Tropical, rainforest | - Bogor | **C) Temperate** | Cfa | Temperate, no dry season, hot summer | - Gwangju |
|  |  |  | - Kisangani |  |  |  | - Philadelphia |
|  |  |  | - Manaus |  |  |  | - Rasht |
|  |  |  | - Mbandaka |  |  |  | - Sydney |
|  | Am | Tropical, monsoon | - Mumbai |  | Cfb | Temperate, no dry season, warm summer | - Curitiba |
|  |  |  | - Mymensingh |  |  |  | - Hamburg |
|  |  |  | - PortoVelho |  |  |  | - Manchester |
|  |  |  | - Riode Janeiro |  |  |  | - Paris |
|  |  |  | - Thrissur |  |  |  | - Vancouver |
|  | Aw | Tropical, savannah | - Belo Horizonte |  | Csa | Temperate, dry summer, hot summer | - Adana |
|  |  |  | - Dhaka |  |  |  | - Barcelona |
|  |  |  | - Surabaya |  |  |  | - Perth |
| **B) Arid** | BSh | Arid, steppe,  hot | - Hargeysa |  | Cwa | Temperate, dry winter, hot summer | - Moradabad |
|  |  |  | - Oran |  |  |  | - Nanyang |
|  |  |  | - Ouagadougou |  |  |  | - Ndola |
|  |  |  | - Shiraz | **D) Cold** | Dfa | Cold, no dry season, hot summer | - Detroit |
|  |  |  | - Tiruppur |  |  |  | - Dnipropetrovs |
|  | BSk | Arid, steppe, cold | - Bloemfontein |  |  |  | - Omaha |
|  |  |  | - Tehran |  |  |  | - Rostovondon |
|  |  |  | - Urmia |  | Dfb | Cold, no dry season, warm summer | - Calgary |
|  | BWh | Arid, desert,  hot | - Khartoum |  |  |  | - Minsk |
|  |  |  | - Las Vegas |  |  |  | - Montreal |
|  |  |  | - Mecca |  |  |  | - Yekaterinburg |
|  |  |  | - Riyadh |  | Dwa | Cold, dry winter, hot summer | - Beijing |
|  |  |  | - Torreon |  |  |  | - Seoul |
|  | BWk | Arid, desert, cold | - Isfahan |  |  |  | - Suwon |
|  |  |  | - Mashhad |  |  |  | - Tangshan |

**
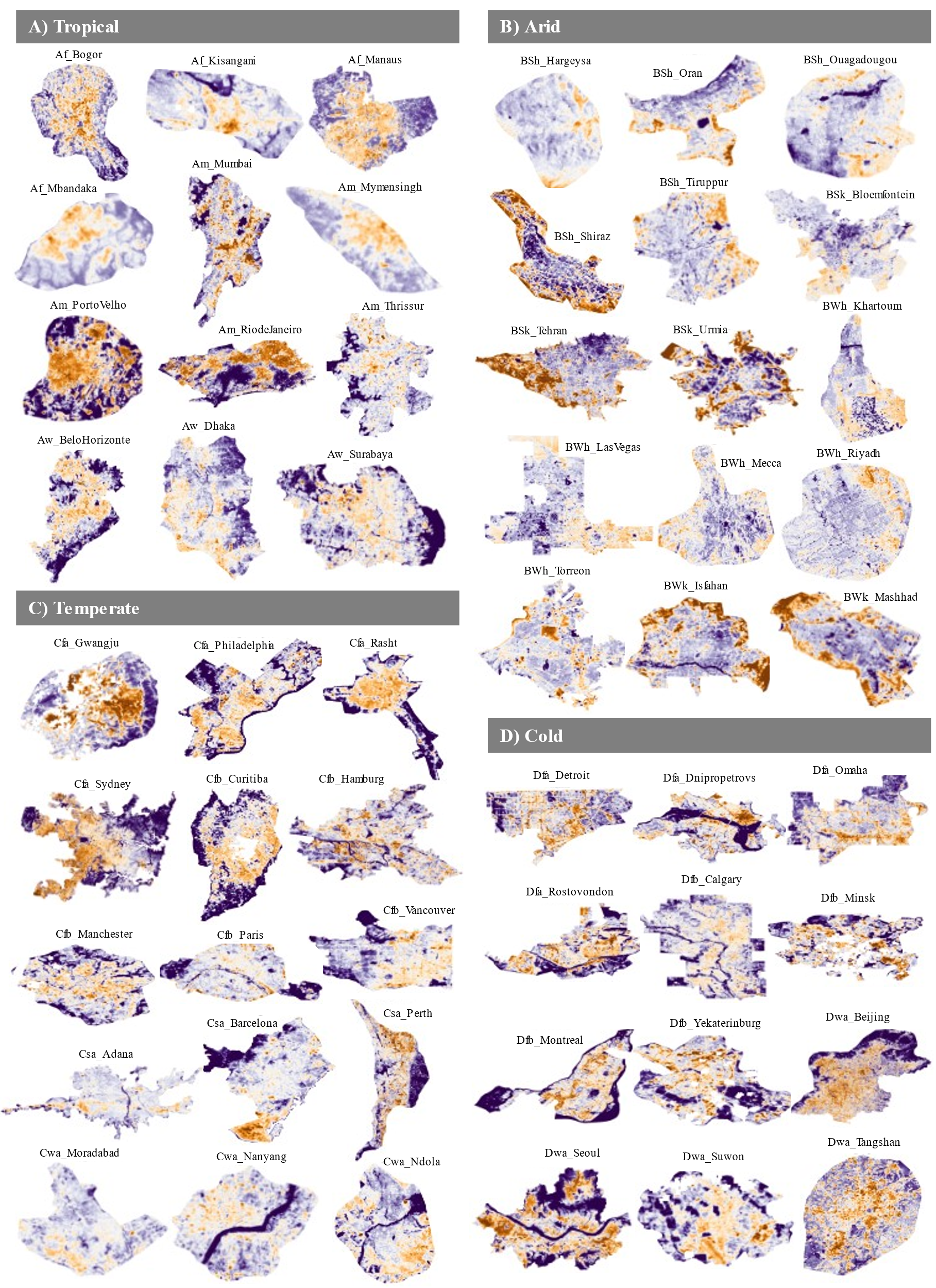
**

**Figure. 1** **Extracted LST maps from Landsat 8 for 54 selected urban areas during warm month (in a year between 2017-2020).** LST was normlised using the median LST value in each city. Colour ranges are the same. These maps were generated in Google Earth Engine platform (<https://earthengine.google.com>) and processed and visualised in Python 3.7.6 using Rasterio (v.1.2.3), NumPy (v.1.18.1), and Matplotlib (v.3.1.3) libraries.

**
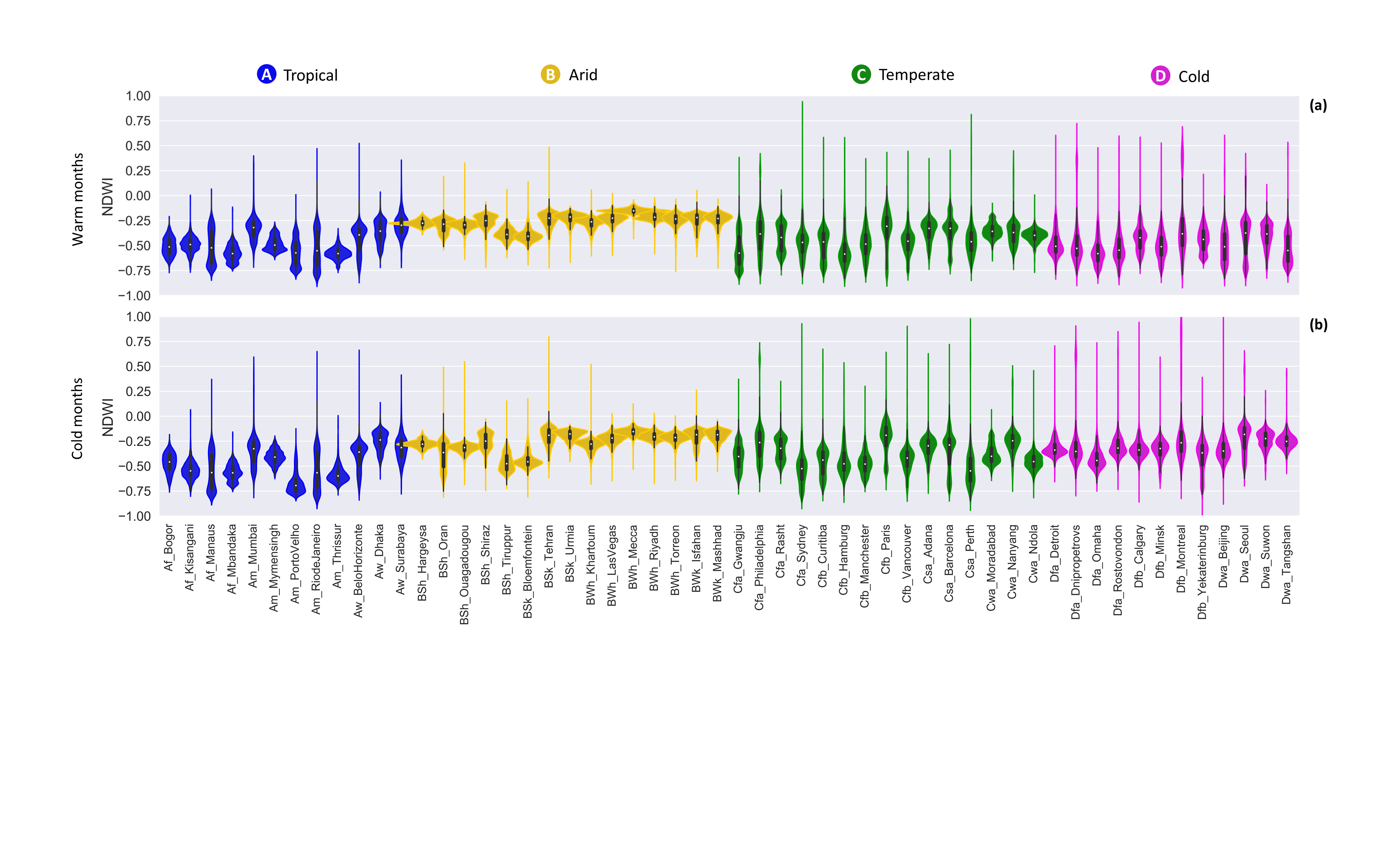
**

**
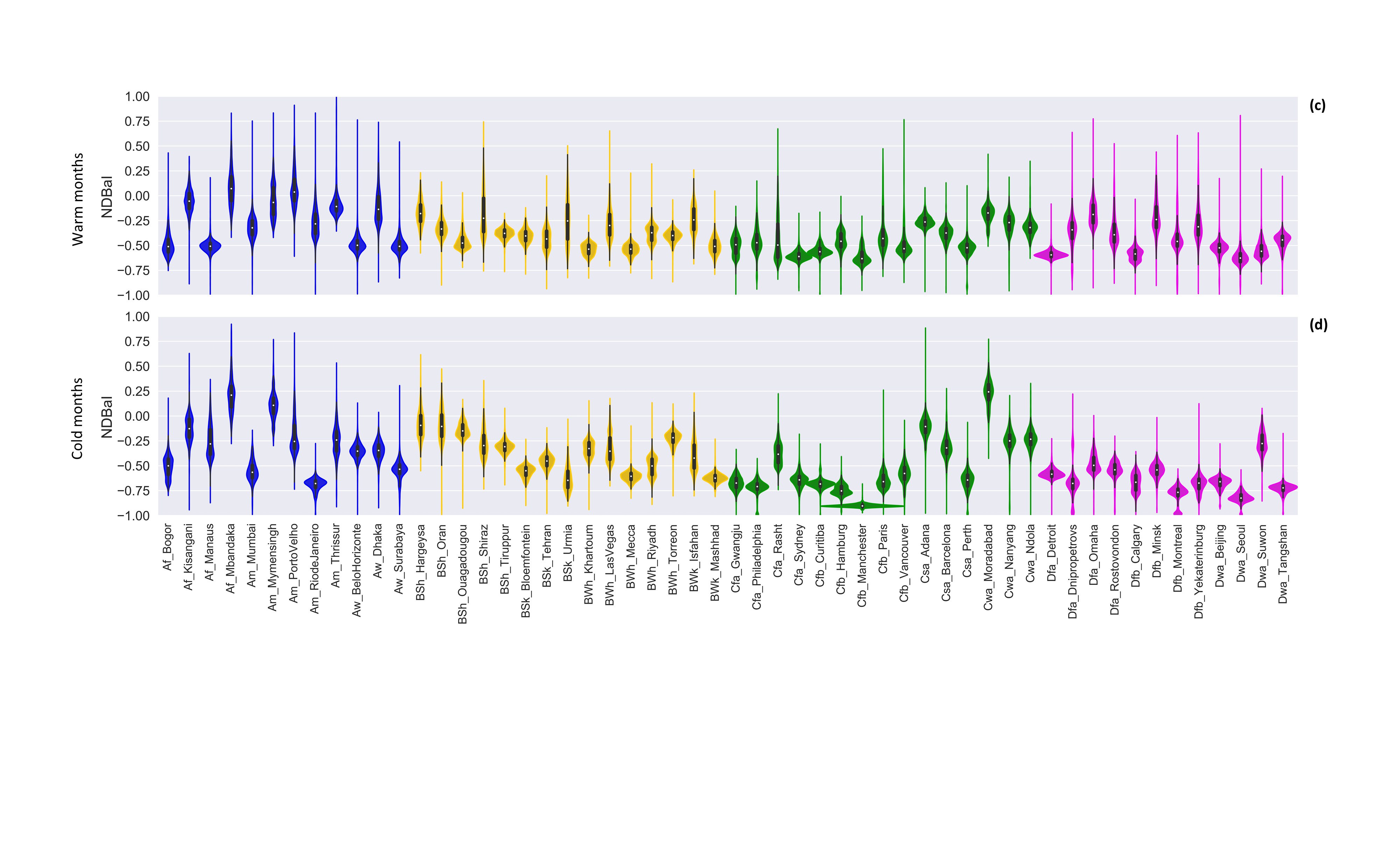
**

**Figure. 2** **Range and distribution of land cover variable values for 54 cities.** Coloured areas represent the distribution of the values. White dots within the violin plots depict the median values. **a** NDWI in warm months. **b** NDWI in cold months. **c** NDBaI in warm months. **d** NDBaI in cold month.


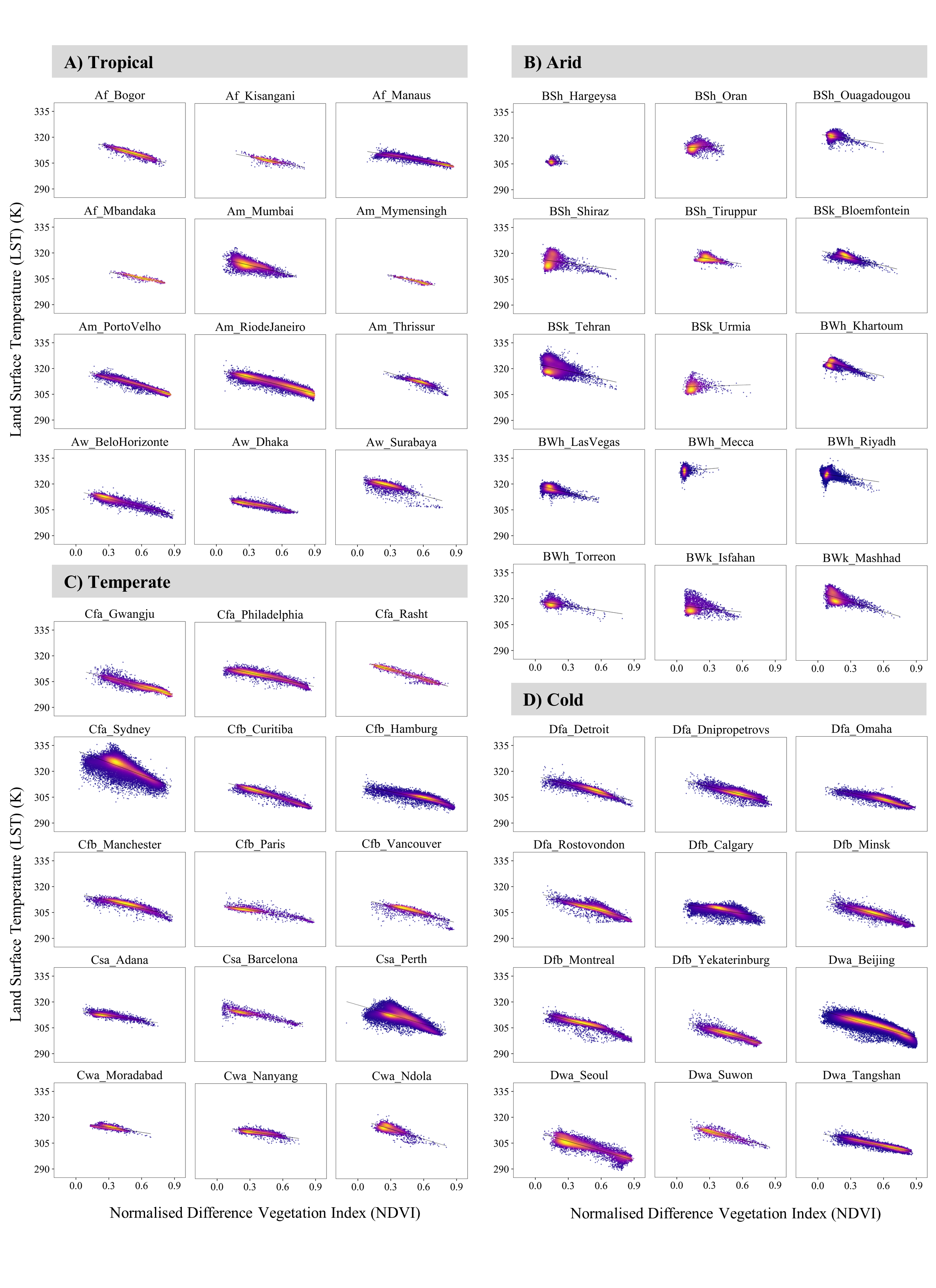


**Figure. 3** **The correlation between NDVI and LST in the cities located in different climate classes during warm months (in a year between 2017-2020).** All the relations are statistically significant at 0.01 level with P-value of 0.000.


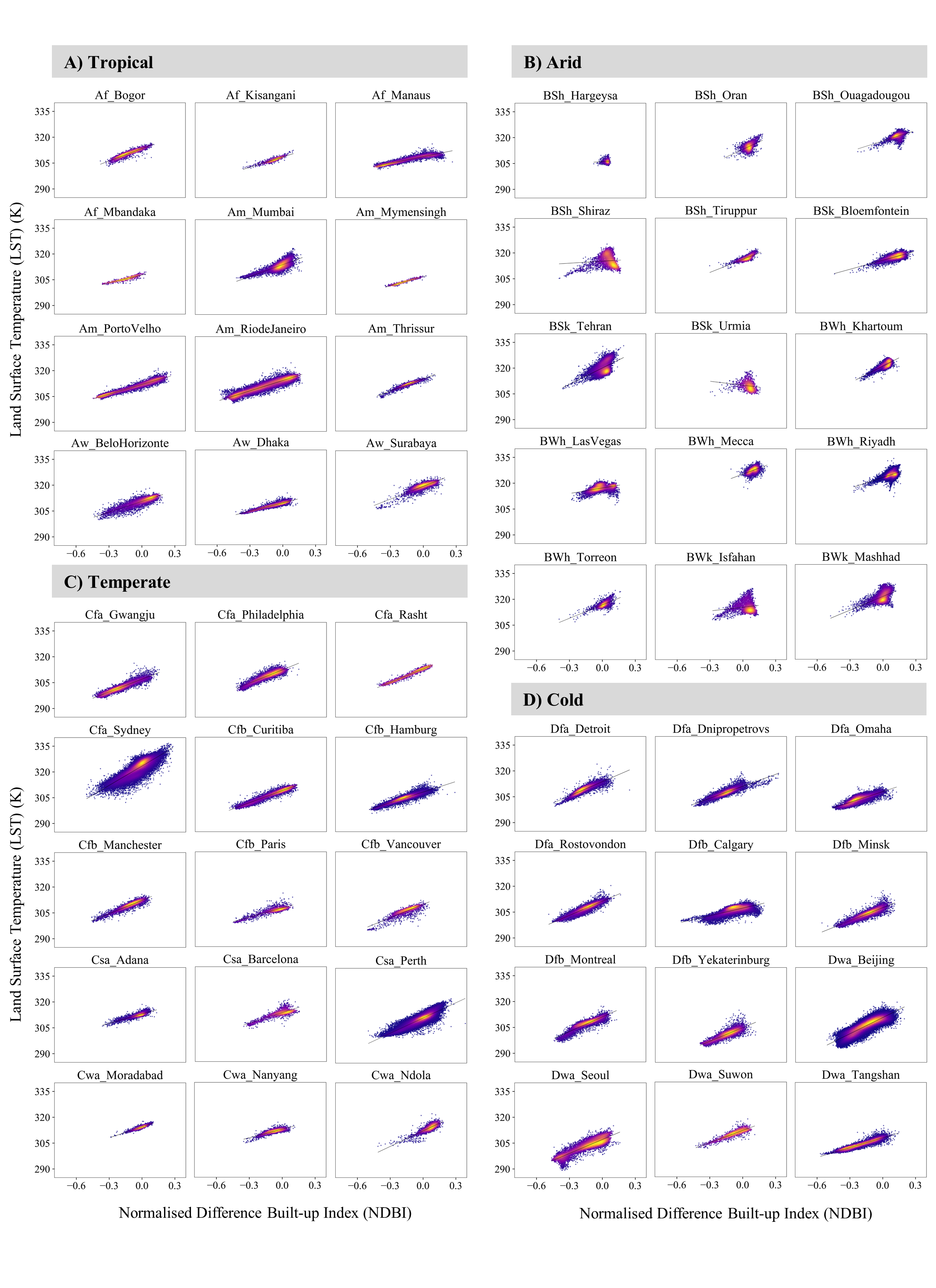


**Figure. 4** **The correlation between NDBI and LST in the cities located in different climate classes during warm months (in a year between 2017-2020).** All the relations are statistically significant at 0.01 level with P-value of 0.000.


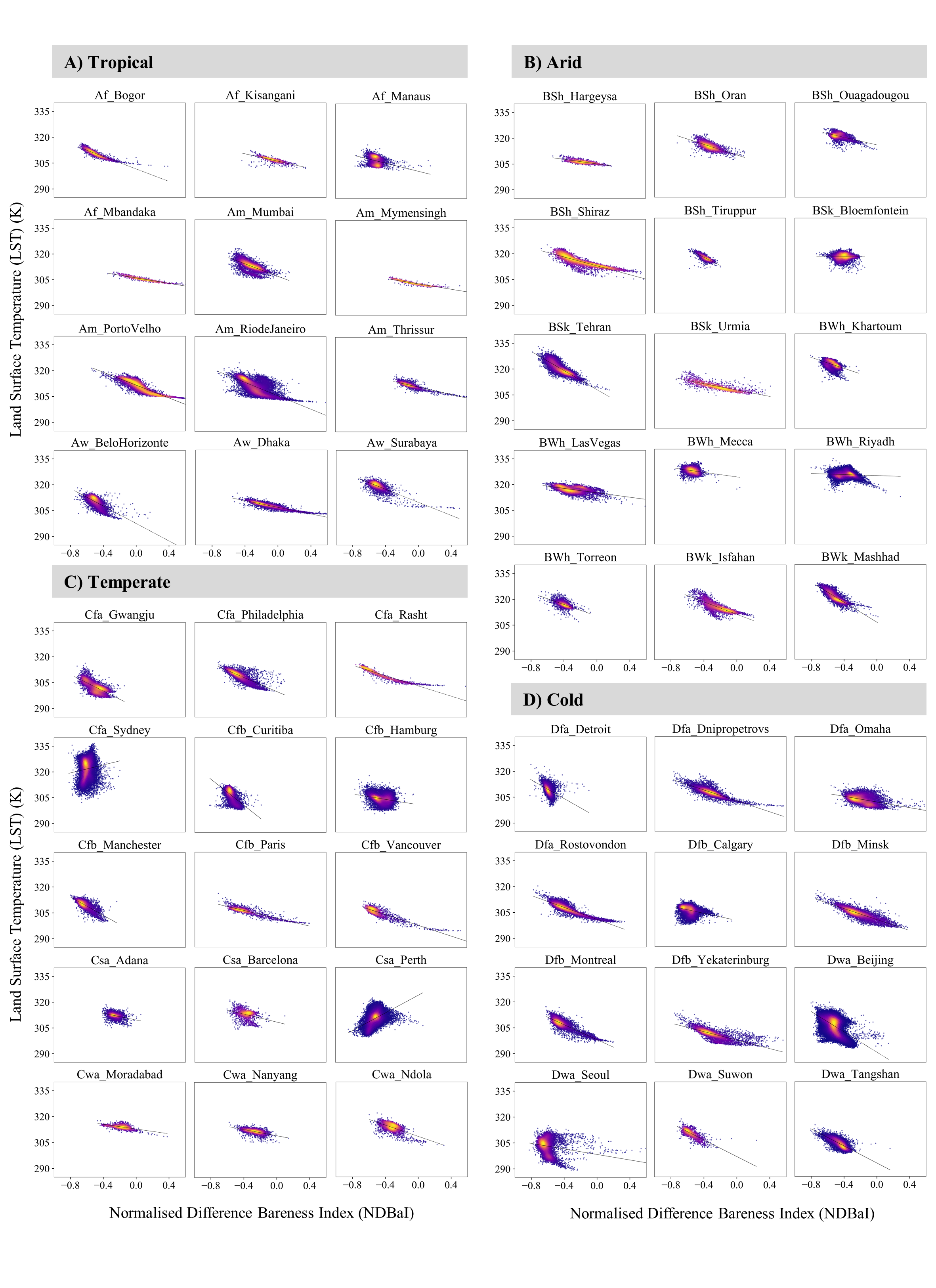


**Figure. 5** **The correlation between NDBaI and LST in the cities located in different climate classes during warm months (in a year between 2017-2020).** All the relations are statistically significant at 0.01 level with P-value of 0.000.

**Table 2** **Gradient boosting model performance before and after classifying climate.** RMSE and adjusted R2 values as the measure of model accuracy (explanatory potential of all variables on LST) and model fit, respectively.

|  | **RMSE** | | **Adjusted R2** | |
| --- | --- | --- | --- | --- |
|  | Warm months | Cold months | Warm months | Cold months |
| All climates | 2.26 | 4.29 | 0.90 | 0.78 |
| A_Tropical | 1.09 | 1.92 | 0.94 | 0.87 |
| B_Arid | 1.29 | 2.01 | 0.93 | 0.91 |
| C_Temperate | 1.49 | 2.61 | 0.88 | 0.80 |
| D_Cold | 1.17 | 1.36 | 0.91 | 0.91 |
